# The Dual Role of Autophagy in Cancer Development and a Therapeutic Strategy for Cancer by Targeting Autophagy

**DOI:** 10.3390/ijms22010179

**Published:** 2020-12-26

**Authors:** Chul Won Yun, Juhee Jeon, Gyeongyun Go, Jun Hee Lee, Sang Hun Lee

**Affiliations:** 1 Stembio. Ltd., Entrepreneur 306, Asan-si 31538, Korea; skydbs113@naver.com (C.W.Y.); jeonj1008@gmail.com (J.J.); 2 Department of Biochemistry, College of Medicine, Soonchunhyang University, Cheonan 31151, Korea; ggy0227@naver.com; 3 Institute of Tissue Regeneration Engineering (ITREN), Dankook University, Cheonan 31116, Korea; junheelee@dankook.ac.kr; 4 Department of Nanobiomedical Science and BK21 PLUS NBM Global Research Center for Regenerative Medicine, Dankook University, Cheonan 31116, Korea; 5 Department of Oral Anatomy, College of Dentistry, Dankook University, Cheonan 31116, Korea; 6 Cell & Matter Institute, Dankook University, Cheonan 31116, Korea; 7 Medical Science Research Institute, Soonchunhyang University Seoul Hospital, Seoul 04401, Korea; 8 Department of Anatomy, BK21FORU Project2, College of Medicine, Soonchunhyang University, Cheonan 31151, Korea

**Keywords:** autophagy, cancer, metastasis, drug resistance, tumorigenesis, cancer stem cells, autophagy modulators

## Abstract

Autophagy is a delicate intracellular degradation process that occurs due to diverse stressful conditions, including the accumulation of damaged proteins and organelles as well as nutrient deprivation. The mechanism of autophagy is initiated by the creation of autophagosomes, which capture and encapsulate abnormal components. Afterward, autophagosomes assemble with lysosomes to recycle or remove degradative cargo. The regulation of autophagy has bipolar roles in cancer suppression and promotion in diverse cancers. Furthermore, autophagy modulates the features of tumorigenesis, cancer metastasis, cancer stem cells, and drug resistance against anticancer agents. Some autophagy regulators are used to modulate autophagy for anticancer therapy but the dual roles of autophagy limit their application in anticancer therapy, and present as the main reason for therapy failure. In this review, we summarize the mechanisms of autophagy, tumorigenesis, metastasis, cancer stem cells, and resistance against anticancer agents. Finally, we discuss whether targeting autophagy is a promising and effective therapeutic strategy in anticancer therapy.

## 1. Introduction

Autophagy is a highly conserved cellular process that maintains cellular homeostasis by the degradation and recycling of damaged or long-lived proteins, misfolded proteins, and damaged and abnormal organelles [1,2,3]. In addition, autophagy is regulated to protect against diverse cellular stress conditions and is involved in starvation, DNA damage, hypoxia, exposure to chemotherapy, and meets the metabolic requirements of cells to retain the function of organelles and cellular signaling pathways. Autophagy is a delicate degradation process induced by double membrane vesicles called autophagosomes, which then form the autolysosomes—after fusion with lysosomes—to recycle cellular components [2,4].

Autophagy is critical for the preservation of health and initiation of diseases. Abnormal autophagy is associated with a variety of diseases, including neurodegenerative disease [5], cardio-related diseases [6], type II diabetes [7], and cancer [8]. Autophagy has a tumor-suppressive role in cancer initiation and the progression of malignant tumors. The inhibitory effects are brought about by the elimination of abnormal cells and organelles and the restriction of cell proliferation and genomic instability in cancer [9,10,11]. In addition, autophagy plays a promoting role in cancer cells by meeting the metabolic demand of proliferating cancer cells and inducing chemoresistance [12,13].

In this review, we summarize several mechanisms of autophagy in cancer biology as well as its roles in tumor processes, such as tumorigenesis, metastasis, and drug resistance. Next, we discuss the function of autophagy in cancer stem cells (CSCs). Finally, we discuss the potential of targeting autophagy as a promising therapeutic strategy for anticancer therapy.

## 2. The Basic Mechanism of Autophagy

**Figure 1:**
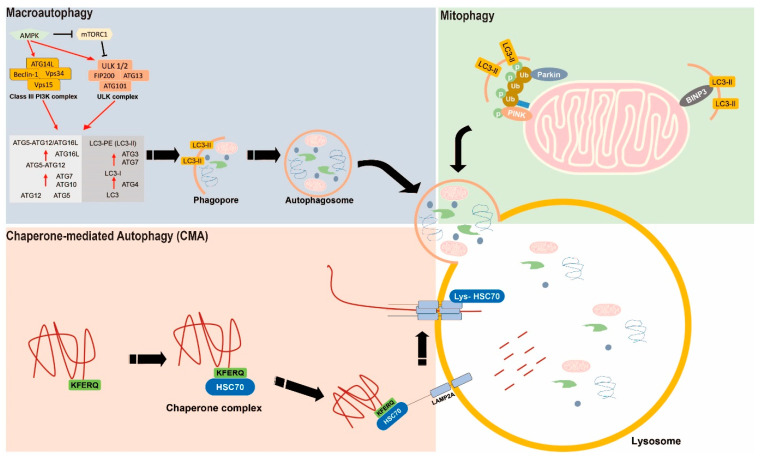
Scheme illustrating the diverse autophagic pathways in cancer cells. Macroautophagy is common autophagic pathway, mitophagy is a specific autophagy, which is working in mitochondria, and chaperone-mediated autophagy is a selective autopahgy with unique mechanism. Black arrows is showed the autophagic pathway. Red arrows is showed the autophagic signal pathway.

Autophagy is a highly evolutionarily conserved process that meets metabolic demands and homeostasis through an intracellular recycling system or self-degradation [14]. The autophagic process is induced under diverse cellular stress conditions, such as starvation, cellular damage, and the production of dysfunctional proteins [8]. Autophagy is classified into macroautophagy, microautophagy, and chaperone-mediated autophagy (CMA). Macroautophagy is involved in the isolation of cytoplasmic cargo into phagophores, which induces the formation of autophagosomes composed of double membrane vesicles. Then, autophagosomes fuse with lysosomes to form autolysosomes and carry out degradation and recycling [15]. Microautophagy is a direct autophagic process involving the invagination of cytosolic components into the lysosomal membrane by the capturing of cargo [16]. CMA is a selective autophagy, in which cargo complexed with chaperone proteins (such as HSC70) is recognized and translocated across the lysosomal lumen by a lysosomal membrane receptor, such as lysosomal-associated membrane 2A (LAMP-2A) (Figure 1) [17].

### 2.1. Macroautophagy

The macroautophagic process comprises various steps, such as initiation, nucleation, and maturation. In the initiation step, several autophagy-related genes (ATGs) are involved in the formation of phagopores, which are derived from mitochondria, endoplasmic reticula, and plasma membranes [18]. The Unc-51-like kinase 1 (ULK1)/ATG13/FAK family-interacting protein 200 kD (FIP200) kinase complex is activated by the inactivation of the mammalian target of rapamycin complex 1 (mTORC1) and activation of the AMP-activated protein kinase (AMPK) [19]. Therefore, the activation of ULK1/ATG13/FIP200 regulates the other ATG proteins and initiates the formation of autophagosomes [20,21]. The membranes of autophagosomes consist of abundant phosphatidylinositol 3-phosphate (PI3P) and are modulated by the class-III phosphatidylinositol 3-kinase complex, which is organized into the vacuolar protein sorting-associated protein 34 (Vps34), Vps15, Atg14, and Beclin1. This complex forms the phagophore assembly site (PAS) [22]. In the maturation step, two ubiquitin-like protein (UBL) conjugation systems, such as ATG12/ATG5/ATG16 UBL and microtubule-associated light chain B (LC3B)-phosphatidylethanolamine (PE) UBL, are required for the PAS for the maturation of autophagosomes, and to induce the elongation of phagopore membranes [23]. ATG12/ATG5 conjugation is organized with ATG16L1 and anchored on PI3P to elongate autophagosomal membranes by WD-repeat domain PI-interacting protein 2 [24]. LC3 is formed into pro-LC3 and cleaved by ATG4. Then, the cleaved LC3 is linked to PE by ATG7 and ATG3 to form LC3-II, which is its lipidated form [25]. Next, LC3-II is allowed to fuse with autophagosomes and lysosomes, and the cytosolic cargo is degraded by lysosomal hydrolases [26].

### 2.2. Mitophagy

Mitochondria are essential cell organelles for metabolism that play a critical role in energy production, cell transcription, cell death control, and homeostasis maintenance [27]. Aging or damaged mitochondria are removed by the autophagocytosis process called mitophagy [28]. Mitochondria with disrupted function are eliminated by mitophagy; new mitochondria are generated and damage to organization and cells is prevented [29]. Signaling pathways that regulate mitophagy can be classified into the two following categories: PTEN-induced putative kinase 1 (PINK1)-Parkin mediated and not mediated [27]. Mitophagy may induce the degradation of damaged mitochondria by the E3 ubiquitin ligase Parkin and the kinase PINK1 [30]. The loss of mitochondrial membrane potential prevents the proteases from degrading PINKl, and the accumulation of PINK1 protein promotes the phosphorylation of Parkin [31]. Activated Parkin promotes the ubiquitination of mitochondrial proteins, and mitophagy induces the degradation of abnormal mitochondrial proteins by the proteasome [32,33]. In addition to the PINK1–Parkin pathway, mitophagy is induced by various proteins, such as NIX, BNIP3, and FUNDC1. These receptors induce mitophagy by interacting with the LC3 protein in the hypoxic state [34,35,36].

### 2.3. Chaperone-Mediated Autophagy

CMA is a selective type of autophagy with unique mechanisms for cargo recognition and translocation into the lysosomal membrane in mammalian cells [17]. CMA works on proteins targeted by heat shock 70 kDa protein 8 (HSC70), which is recognized and bound to the pentapeptide motif (KFERQ) in the substrate protein [37]. HSC70-linked substrate proteins move to the lysosomal membrane and bind to the monomer of the cytosolic tail of LAMP2A and induce the multimerization of LAMP2A [38,39]. The substrate proteins are unfolded and translocation into the lysosomal lumen is mediated, and these proteins are quickly degraded. In addition, substrate–receptor complexes are stabilized by HSP90 on the lumenal side of the lysosomal membrane [38]. After translocation of the substrate by lumenal HSC70, these complexes are separated by cytosolic HSC70, and LAMP2A is reverted to its monomeric form and newly binds the substrate proteins and commences translocation [38]. Therefore, modulation of the CMA process rate is related to the expression levels of LAMP2A, and regulates CMA activity and the degradation of substrate proteins [40].

## 3. The Bipolar Role of Autophagy in Cancer

**Figure 2:**
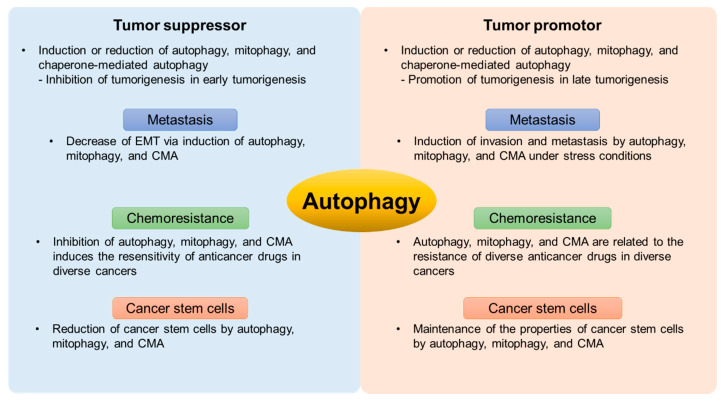
Schematic diagram of the autophagic roles of tumor promotion and suppression in cancer cells.

Autophagy works to maintain cellular homeostasis and degrade damaged proteins and organelles. In addition, many studies have suggested that autophagy is related to an important role in several diseases, although it is currently unclear whether autophagy plays a protective or inhibitory role [41]. In cancer, autophagy has a dual role in tumor promotion and suppression. Autophagy has an inhibitory function through the elimination of damaged cells and organelles during tumor initiation and malignant transformation [42]. In addition, autophagy has a protective function by meeting the metabolic and energy production demand of cancer cells during cancer development (Figure 2) [43].

### 3.1. The Mechanism of Autophagy in Tumorigenesis

Autophagy has a suppressive role in tumorigenesis due to the preservation of physiological homeostasis [44]. In addition, autophagy prevents the conversion of normal cells to malignant cells by decreasing genotoxic stress and inflammation related to tumorigenesis [45]. The inhibition or deficiency of autophagy promotes oncogenesis and malignant transformation. A relationship between autophagy and tumorigenesis has been found through the ATG knockout (KO) animal model, with results showing roles of Beclin-1, ATGs, and inhibition of autophagy in inducing a high incidence of carcinogenesis [46,47].

Beclin-1 is related to the initiation of autophagy and several other cellular processes, such as development, aging, adaptation to stress, and cell death. Additionally, Beclin-1 modulates cancer initiation and progression by regulating autophagic activity by interacting with other autophagy mediators, such as ATGs, mTOR, PI3K-III, and P53 [48]. Beclin-1 regulates autophagic activity toward the suppression of tumorigenesis and a decrease in its expression causes cancer proliferation and tumorigenesis [49]. Beclin-1 induces the autophagy and inhibits human epidermal growth factor receptor 2 (HER2)-mediated tumorigenesis. On the other hand, HER2 binds the Beclin-1 and suppresses the autophagy, and then induces the tumorigenesis of breast cancer cells [50]. Moreover, ABHD5 (abhydrolase domain containing 5) shows the tumor suppressive role in colorectal cancer and regulates the autophagy and CRC tumorigenesis via interaction with Beclin-1 [51]. The BECN1 gene is rarely mutated in cancer; however, the mutation of BECN1 occurs in colorectal, gastric, breast, and prostate cancer [52] due to the similar genomic proximity to the breast cancer susceptibility gene BRCA-1 [53]. The inhibition of Beclin-1 induces the reduction of autophagic activity in hepatocellular carcinoma (HCC), with a tendency to initiate the voluntary formation of malignant lesions in Beclin-1 heterozygous disruption mouse models [54].

The expression level of p62 is related to cancer development [55,56]. p62 is associated with the activity of autophagy as a substrate protein and decreases autophagy through its accumulation in cells. Moreover, p62 plays a protective role in cells by preventing cellular stress through a variety of signaling pathways, such as the interaction of kelch-like ECH-associated protein 1/nuclear factor erythroid 2-related factor 2 (Nrf2) [57] and tumor necrosis factor receptor-associated factor 6/receptor-interacting serine/threonine-protein kinase-1 and mammalian mitogen-activated protein kinase/regulatory-associated protein of mTOR [58]. Therefore, increased p62 concentration contributes to cancer cell survival and enhances tumor progression. p62 is activated to Nrf2 and induces the increased potential of cell proliferation and anticancer drug resistance in HCC cells [57]. Moreover, the accumulation of p62 is observed in clinical liver tumors, and the loss of p62 is observed in liver-specific ATG5 or ATG7-deficient mice [55]. p62 overexpresses in nasopharyngeal carcinoma (NPC) and related to tumor invasion and metastasis. The inhibition of p62 shows the decreased proliferation, clone forming ability, autophagy, and migration via regulation of ERK pathway, and NPC clinical analysis indicates the relation with p62 and metastasis [59]. The expression of p62 decreases by programmed cell death 4 (PDCD4), and suppresses the cell proliferation and tumorigenesis and induces the apoptosis in lung cancer cells [60].

Mitophagy is the specific and selective autophagy of mitochondria and plays an important role in mitochondrial homeostasis and quality control through the elimination of damaged and abnormal mitochondria [61]. Deficiency of mitophagy is related to the damage of mitochondrial functions, tumorigenesis, and tumor progression in various cancers [62]. The function of mitophagy includes a variety of roles, depending on the stage of tumor progression. In early tumorigenesis, mitophagy maintains the metabolic demand of normal cells and suppresses tumorigenesis. In late tumor development, mitophagy enhances cell tolerance and improves tumor progression [63]. The loss of mitochondrial function by mutation or functional change in a variety of critical genes leads to the accumulation of impaired mitochondria and the inhibition of mitophagy, thus, the induction of tumorigenesis [64]. The Pink1/Parkin signal pathway has a key role in the mitophagy pathway [65]. The decreased function of Parkin suppresses mitophagy and induces oncogenesis in various cancer models. The suppression of mitophagy leads to increased reactive oxygen species (ROS) levels, which influences the function of cells and organelles. In Parkin KO mouse models, the loss of Parkin induces the voluntary development of HCC [66,67].

CMA is related to selectively degrade targeted proteins and participates in the modulation of several cellular processes. Increased CMA induces the protoncogenic and prosurvival functions in many cancers. CMA can control the expression levels of specific proteins, such as proto-oncogenic proteins [double-minute 2 homolog (MDM2)] and translationally controlled tumor-associated protein [68,69]. In addition, CMA limits the malignant transformation of normal cells. Abnormal CMA is related to aging and is a high-risk factor for various cancers. The inhibition of CMA in mouse livers leads to a higher incidence of voluntary hepatic tumors with age [70]. Moreover, CMA is activated by lipids, and increased LAMP2A levels are observed in non-alcoholic steatohepatitis mouse models [71,72]. In contrast, the irregular increased intracellular lipids suppress CMA and induce the reduction of LAMP2A expression in steatosis models [73,74]. Decreased CMA induces steatosis, and this disease further suppresses CMA. Eventually, the inhibition of CMA leads to the perpetuation of metabolic dysregulation and progression to fibrosis and HCC [74]. Moreover, sorting nexin 10 (SNX10) relates to the activation of CMA by regulating expression of p21^Cip1/WAF1^ and the deficiency of SNX10 induces the tumorigenesis and progression of colorectal cancer via activation of CMA (29355659). Increased CMA activity induces the tumorigenesis and metastasis of human breast cancer cells via downregulation of ATG5-mediated macroautophagy. The suppression of CMA activity inhibits the tumor growth and metastasis by downregulation of LAMP2A in breast cancer cells in vivo and in vitro [75].

### 3.2. The Relationship between Autophagy and Metastasis

Tumors can locally invade and cause distant metastasis, which causes over 90% of cancer deaths. Recently, proportions of cancer deaths caused by metastases was determined [76]. In this study, the population-based data from the Cancer Registry of Norway for the years 2005-2015 was analyzed. The results showed that, for solid tumors, 66.7% of cancer deaths were registered with metastases as a contributing cause. In addition to cancer metastasis, other causes of death may due to side effects from systemic cancer treatment, or some patients commit suicide after receiving cancer diagnosis [77]. Tumor cells can escape from primary tumors by invasion and metastasis under stressful conditions, such as starvation and hypoxia. In primary cancer, autophagy is related to hypoxia and starvation, and the inhibition of tumor cell necrosis and inflammation [78]. Moreover, autophagy suppresses the epithelial–mesenchymal transition (EMT) through the degradation of p62 and the cargo protein TWIST1, which promotes EMT as a transcription factor [79]. Autophagy can enhance or reduce the invasion and metastasis of cancer. During initial metastasis, autophagy enhances the survival of cancer cells through metabolic stress and hypoxia by decreasing necrosis [80,81]. In addition, autophagy diminishes the infiltration of macrophages that is required for the start of metastasis [82,83,84], and autophagy can enhance the modulation of cell adhesion signaling pathways and promote cancer invasion and migration [85]. Autophagy leads to the overcoming of cell death by anoikis, which is a type of cell death induced by the detachment from the extracellular matrix [86,87,88].

The specific inhibition of focal adhesion kinase activates the SRC kinase and suppresses autophagy [89]. Moreover, the suppression of autophagy inhibits cell migration and metastasis by regulating oncogenic SRC activity through interactions with LC3 and paxillin [85]. Starvation-induced autophagy leads to the induction of metastasis and invasion in HCC cells through the regulation of TGF-β/Smad3 signaling [90]. In addition, the activation of autophagy enhances tumor metastasis and glycolysis through the Wnt/β-catenin signaling-mediated upregulation of monocarboxylate transporter 1 [91]. The inhibition of autophagy by miR-140-5p induces a decrease in cancer cell survival and invasion potential [92]. In addition, the induction of autophagy enhances the degradation of Snail and suppresses EMT and metastasis by decreasing the levels of EMT and metastatic proteins in cancer cells. In contrast, ATG7 knockdown induces the suppression of autophagy, inhibits Snail degradation, and promotes EMT and metastasis [93].

Cancer cells replacing the aerobic respiration of mitochondria with cytosolic lactic acid fermentation is called the Warburg effect, which maintains the energetic demand of cancer cells [94]. Cancer cells exhibit the Warburg effect, dysfunctional quality control of mitochondria, dysfunctional regulation of ROS and the redox state, and deficiency of apoptosis signals [64]. Mitophagy can enhance cancer cell survival by adapting to stress through the elimination of abnormal mitochondria [95,96]. Parkin is a key mediator of mitophagy and inhibits cancer migration and invasion by targeting HIF-1α for ubiquitination and degradation [97]. On the other hand, Parkin is overexpressed in melanoma compared with normal dermatic tissues, and increased Parkin levels induce metastasis and tumor growth, and the loss of Parkin suppresses tumor formation and metastasis through the inhibition of MFN2 ubiquitination [97,98]. BNIP3 is a pro-apoptotic protein that inhibits the fusion of impaired mitochondria and promotes mitophagy [99]. The inhibition of BNIP3 induces abnormal mitophagy and the accumulation of mitochondrial ROS levels as well as enhances cancer metastasis in human triple-negative breast cancer [100]. In contrast, the increased expression of BNIP3 induces excessive mitophagy and suppresses metastasis in HCC cells [101].

CMA is related to cancer metastasis as the inhibition of CMA decreases metastasis through the reduction of migration and resistance to anoikis [102]. There is a correlation between CMA activity and metastasis in breast cancer [75]. CMA degrades the multifunctional protein HSD17B4, which modulates the properties for the invasion and migration of cells [103]. Moreover, decreased CMA by the modulation of LAMP2A inhibits tumor growth and metastasis by upregulating ATG5-dependent macrophages in human breast cancer [75].

### 3.3. The Roles of Autophagy in Chemoresistance

Anticancer therapy, such as the use of chemotherapeutic agents, induces cancer cell apoptosis. However, multi-drug resistance (MDR) can occur with prolonged exposure to the same drugs combined with the deficiency of apoptosis [104]. Autophagy plays a protective role by removing the damaged organelles and proteins, and excessive autophagy plays a suppressive role by inducing autophagic cell death. Further, autophagy contributes to tumorigenesis, cancer progression, and resistance to anticancer therapy [105]. Autophagy is induced during anticancer therapies, such as radiation therapy, chemotherapy, and targeted therapies, predominantly through cytoprotective functions through the induction of MDR against therapy-induced stress responses [106,107,108]. Therefore, the suppression of autophagy resensitizes cancer cells and promotes the therapeutic effects of anticancer therapies. In contrast, autophagy mediates autophagic cell death, which differs from apoptosis.

MDR in cancer is induced by diverse factors, such as heterogeneity, target mutation, and the cancer microenvironment [109,110,111]. Increased autophagy induces MDR and is related to a poor prognosis. S100 calcium-binding protein A8 (S100A8) induces the development of MDR through the regulation of autophagy via interactions with S100A8 and BECN1 in leukemia cells [112]. HSP90AA1 is induced by chemotherapeutic reagents, such as doxorubicin, cisplatin, and methotrexate, in osteosarcoma. The inhibition of HSP90AA1 restores the sensitivity to chemotherapy in osteosarcoma cells through the reduction of autophagy via the PI3K/AKT/mTOR pathway and the induction of apoptosis via the JNK/P38 pathway [113]. CircPAN3, which is a circular RNA, mediates the development of acute myeloid leukemia (AML) drug resistance, and the inhibition of cricPAN3 resensitizes the drug resistance to doxorubicin by modulating autophagy, which is regulated by the AMPK/mTOR pathway, and inducing apoptosis-related proteins in ADM-resistant cells [114]. Poly (adenosine diphosphate ribose) polymerase (PARP) inhibitors have anti-cancer activity against ovarian cancers, but their therapeutic efficiency is restricted by drug resistance. PARP inhibitors, such as olaparib, niraparib, rucaparib, and talazoparib, are limited in usage due to this acquired drug resistance by the upregulation of autophagy in ovarian cancer cells and the inhibition of autophagy with chloroquine (CQ) promotes the sensitivity of ovarian cancers [115]. The inhibition of autophagy using CQ restores the sensitivity to paclitaxel and decreases the potential of metastasis of NSCLCs via an increase in the levels of intracellular ROS and modulation of the Wnt/β-catenin signaling pathway and AKT activity [116]. 3-methyladenine (3-MA) is an autophagy inhibitor and treatment with 3-MA resensitizes the resistance of CDDP-resistant osteosarcoma cells against chemotherapy agents. The suppression of autophagy by 3-MA enhances the expression of FOXO3A transcription factor and PUMA, which is a pro-apoptotic protein, and leads to an increase in apoptosis [117].

miRNAs can contribute to resistance to anticancer therapy. miR-496-3p acts as a tumor suppressor or oncogene miRNA in cancer, and is related to cancer proliferation, metastasis, and chemoresistance. Decreased expression of miR-495-3p occurs in gastric cancer tissue and MDR cell lines and the overexpression of miR-495-3p restores the sensitivity of MDR gastric cancer to four chemotherapeutic agents (adriamycin, CDDP, fluorouracil, and vinscristine) [118]. On the other hand, decreased miR-495-3p inhibits the tumor growth in vivo via the suppression of autophagy by downregulating GRP78, which stimulates AMPK and activates class III PI3K. The low expression level of miR-30a induces chemoresistance against cisplatin and weakens apoptosis in gastric cancer [119]. The upregulation of miR-30a restores the sensitivity to cisplatin in resistant gastric cancer by reducing autophagy and promoting apoptosis. The expression of miR-199a-5p is decreased in patients with AML and adriamycin-resistant AML cells. The inhibition of miR-199a-5p restores the sensitivity to adriamycin in AML by regulating autophagy via the inhibition of the damage regulator autophagy modulator 1 [120]. Overexpressed miR-22 induces the resensitization of 5-FU-resistant colorectal cancer through the inhibition of autophagy and increase in apoptosis, via the downregulation of B-cell translocation gene 1 [121]. Moreover, miR-26a reduces dabrafenib-mediated autophagy and promotes the sensitivity of melanoma cells to dabrafenib by targeting HMGB1 [122].

Mitophagy is related to the efficacy of chemotherapy and acquisition of drug resistance. Mitophagy rapidly removes the impaired mitochondria—resulting from the chemotherapeutic treatment—of cancer cells and mediates drug resistance [123]. General chemotherapeutic drugs, including cisplatin, paclitaxel, doxorubicin, and 5-fluorouracil, remove cancer cells in the anticancer therapy of several solid cancers. However, the acquisition of drug resistance induces the failure of anticancer therapy by autophagy or mitophagy [124]. The E3 ubiquitin ligase ARIH1 is widely expressed in many cancers, such as breast cancer and lung adenocarcinoma, and induces mitophagy in a PINK1-dependent manner. ARIK1-mediated mitophagy inhibits chemotherapy-induced cell death and leads to acquired chemoresistance [125]. The inhibition of PINK1–Parkin mediated mitophagy enhances the efficacy of the anticancer drug B5G1, a new derivative of betulinic acid, by inducing cancer cell death [126]. The induction of mitophagy using BAY 87-2243, an inhibitor of mitochondrial respiratory chain complex I, enhances necrosis and ferroptosis by increasing ROS levels in melanoma cells [127]. The suppression of mitophagy using liensinine as a major isoquinoline alkaloid restores the sensitivity of doxorubicin in breast cancer and promotes doxorubicin-induced apoptosis through the induction of DNM1L-mediated mitochondrial fusion [128].

### 3.4. Autophagy in Cancer Stem Cells

CSCs (also known as tumor-initiating or tumor-propagating cells) are characterized by limited self-renewal and differentiation abilities compared to normal stem cells [129]. Pluripotency is the main feature of CSCs, and they can divide and maintain an undifferentiated state indefinitely. CSCs were first isolated by John Dick’s group using fluorescence activated cell sorting based on CD34 and CD38 (CD34+CD38-) surface marker expression in AML [130]. Autophagy is a major factor in the survival and resistance of CSCs [131,132]. Autophagy plays a critical role in maintaining dynamic equilibrium between CSCs and cancer cells [133]. In particular, the protein/organ quality control of autophagy may be relevant during periods of quiescence and/or differentiation [134]. The delicate mechanism of autophagy in the biology of CSCs has not yet been explained. Considering the similarity between CSCs and stem cells, it is expected that autophagy plays a protective role in CSCs. Nevertheless, deregulation of this catabolic process in CSCs may also be justified, as autophagy suppresses the early stages of tumorigenesis. We summarize below the experimental results for autophagy in CSCs.

Autophagy is associated with various CSCs [45], such as colon [135,136], breast [137,138], pancreatic [139,140], AML [130,141], ovarian [142], and glioblastoma [143] CSCs, and impairment of autophagy negatively influences the expression of stemness markers and cell self-renewal capacity. In colon CSCs, curcumin induces the survival of colon CSCs and significantly decreases the expression levels of stemness markers at the optimal concentration. Curcumin enhances proliferation and reduces autophagic cell death in CSCs. Spheroid cultures are degraded by curcumin in vitro but are regenerated within 30 to 40 days after treatment with cisplatin [144]. This suggests the survival benefits of autophagy while allowing the long-term persistence of colon cancer.

Studies regarding breast CSCs have clarified that autophagy homeostasis is an essential function for maintaining pluripotency under various pathophysiological conditions [145]. Compared to adherent cells, autophagy is upregulated in mammospheres, and the key proteins BECLIN1 and ATG4, which are involved in autophagy, are necessary for maintenance and expansion [146,147]. Serum-deprived mesenchymal stem cells (SD-MSCs) assist MCF-7 tumor growth, and SD-MSC-injected tumors show differentiation and decreased apoptosis. The staining of Beclin-1 reveals autophagic regions surrounding active proliferating cells. Furthermore, SD-MSCs survive via autophagy and release paracrine factors that assist tumor cells under nutrient/serum deficiency [133]. Autophagy markers, such as Atg5, Atg12, and LC3B, are overexpressed in dormant stem cells, such as breast cancer cells, and the suppression of autophagy by 3-MA reverses dormant expression [148]. c-Jun NH2 terminal kinase (JNK/SAPK) is unregulated in breast cancer cells, such as dormant stem cells, and is responsible for enhancing autophagy [138]. Increased expression of Beclin1 is found in human breast cancer as well as other breast cancer cell lines, such as MCF-7 and BT474, which are crucial for Beclin1 to maintain CSCs and tumor development [137].

Higher autophagic flux has revealed the increased expression of HIF-1α and its specific role in promoting dynamic equilibrium between CSCs and non-CSCs [149], which is important for developing a treatment strategy that targets CSCs as well as microenvironmental impacts on tumors. In hematologic malignancies, autophagy can act as both a chemoresistance and tumor-suppressive mechanism. Depending on the type of autophagy and the stage of the leukemia (early versus advanced), autophagy can play opposite roles. The autophagy level in CML appears to be closer to that of solid tumor CSCs, as some ATGs (ATG4, ATG5, and BECLIN 1) are upregulated and the inhibition of ATG7 or ATG4B by small interfering RNA (siRNA) influences cell survival [150,151,152]. In contrast, the function of autophagy is necessary to protect the progression of myelodysplastic syndrome. Many ATGs will mutate or downregulate in some patients with AML. The suppression of ATGs enhances apoptosis caused by imatinib mesylate (IM) in CML cell lines and primary CML cells. Phenotypically and functionally defined CML stem cells are completely eliminated by combination treatment with tyrosine kinase inhibitors (TKIs), such as IM, nilotinib, or dasatinib, and an autophagy inhibitor [131]. These results indicate that autophagy inhibitors promote the therapeutic effect of TKIs in CML treatment.

Side population (SP) is a subset of CSCs, and the SP of urinary bladder cancer cells (T25, UM-UC-3) shows a high mRNA expression of stemness genes. In addition, these cells are likely to create rotational force in non-adherent conditions in comparison with non-SP and other cells. SP cells exhibit significant resistance against gemcitabine, mitomycin, and cisplatin treatment. In addition, SP cells, depending on the autophagic flux rate, show resistance to chemotherapy, and the inhibition of autophagy through pharmacological reagents and siRNA, promotes the therapeutic effects of bomitabine, mitomycin, and cisplatin in SP cells [153]. In contrast, delat-24-RGF, an antimicrobial agent for glioblastoma cells, induces the accumulation of autophagic proteins and vacuoles in brain CSCs isolated from surgical glioblastoma, and this accumulation promotes apoptosis [132]. In glioblastoma mouse models, the treatment of delta-24-RGD improved survival, and immunofluorescence analysis has shown that the expression of ATG increases and can be used as a marker for the glioblastoma prevention effect.

The fate of autophagy appears to differ based on diverse factors, such as stimulus, cell type, and microenvironment. Therefore, understanding the mechanism of autophagy is critical for determining its role in CSCs and developing therapeutic strategies. Further studies are needed regarding novel and promising autophagy regulators for more effective and safer anticancer strategies.

## 4. Targeting Autophagy as an Anticancer Therapy

### 4.1. The Effect of Autophagy Inhibitors in Anticancer Therapy

The upregulation of autophagy is related to the acquired mechanism of drug resistance and survival of cancer cells [104]. The suppression of autophagy by genetic or pharmacological methods enhances the sensitivity of anticancer therapy in diverse cancer cells [154]. Diverse inhibitors of autophagy can be used alone or in combination with anticancer drugs for anticancer therapy (Table 1).

The inhibition of autophagy by ATG5 and Beclin-1 siRNA restores the sensitivity to cisplatin in lung cancer cells and enhances cisplatin-mediated apoptotic cell death by upregulating caspase activity and reducing cell viability [155]. The suppression of autophagy by 3-MA (an autophagy inhibitor) enhances hypoxia-mediated apoptosis in colorectal cancer [9]. Moreover, in bladder cancer, the efficacy of enzalutamide (ENZ) is restricted by the resistance ensued by the induction of autophagy via an increase in AMPK, ATG5, LC3B, and ULK1 levels [9]. Genetic inhibition of autophagy using ATG5 siRNA promotes the ENZ-mediated apoptosis, and the combination treatment with ENZ and CQ improves the therapeutic efficacy by restoring the sensitivity against ENZ via the reduction of tumor growth and induction of apoptosis [156].

Atorvastatin (ATO) is a cholesterol reducing agent that has anticancer effects in diverse cancer cells. ATO decreases cancer growth and enhances apoptosis in cervical cancer cells via the induction of apoptosis-related proteins, such as caspase-3, PARP, and Bim. ATO treatment also induces autophagy, which restricts the therapeutic effect of anticancer drugs. A combination of ATO and an autophagy inhibitor, such as 3-MA or bafilomycin A1 (Baf A1), has synergetic effects, such as the enhancement of ATO-induced apoptosis in cervical cancer [157]. Reactivation of p53 and induction of tumor cell apoptosis (RITA) is a small molecule that interrupts the p53–MDM2 interaction and shows anticancer effects by inducing exclusive apoptosis, but resistance is a major challenge in cancer therapy. The combination treatment with RITA and 3-MA shows effective therapeutic effects of cisplatin- or RITA-resistant head and neck cancer cells via the inhibition of autophagy and induction of apoptosis [158].

Baf A1 exhibits autophagy inhibition and enhancement of apoptosis in the treatment of cancer. However, the therapeutic effect is shown at high concentrations of Baf A1, and its application is limited due to the potential toxicity [159]. Baf A1 has therapeutic effects at low concentrations in pediatric B-cell acute lymphoblastic leukemia via the inhibition of autophagy, targeting of mitochondria, and induction of apoptosis [160]. Cisplatin induces the activation of autophagy, which in turn leads to resistance to cisplatin in bladder cancer [161]. Treatment with Baf A1 promotes the therapeutic effect of cisplatin by inhibiting autophagy. In gastric cancer, resistance to 5-FU treatment is acquired due to autophagy, and combination treatment of Baf A1 inhibits the viability, clone formation, invasion, and migration as well as promotes apoptosis and overcomes 5-FU resistance via the suppression of autophagy [162].

The antimalarial drugs Chloroquine (CQ) and hydroxychloroquine (HCQ) have potential anticancer effects by suppressing autophagy and inducing apoptosis in bladder cancer cells [163]. Recombinant *Bacillus caldovelox* arginase mutant (BCA-M) has been developed for the therapy of several cancer cell lines, and has positive effects in anticancer therapy by reducing cancer growth in human cervical cancer cells [164]. In a phase III clinical trial, BCA-M showed positive therapeutic effects on cancer cells via the inhibition of growth, by increasing apoptosis and cell cycle arrest. In addition, combination treatment with BCA-M and CQ promotes the therapeutic effects of BCA-M by reducing autophagy. In phase I clinical trial, the combination of HCQ and chemotherapeutic reagents is increased the median progression-free survival (mPFS) and overall survival (OS) in 18 patients with relapsed or refractory multiple myeloma [165]. Additionally, the therapeutic efficacy of combination with HCQ and vorinostat (VOR), which is the histone deacetylase inhibitor, has been investigated in 19 patients with metastatic colorectal cancer [166]. The combination treatment is showed the 2.8 months mPFS and 6.7 months OS in patients with refectory colorectal cancer and confirmed the safe and well tolerated in refractory CRC patients. In phase 1/2 trial, 35 patients with borderline resectable pancreatic adenocarcinomas were treated with an HCQ dose of 1200 mg daily until the day of surgery combined with doses of fixed-dose gemcitabine (1500mg/m^2^) [167]. The trial demonstrated that pre-operative autophagy inhibition with HCQ plus gemcitabine is safe and well-tolerated. Nineteen of 35 patients showed a decrease in surrogate biomarker response and 29 of 35 patients underwent surgical resection suggesting autophagy inhibition with HCQ could produce positive outcome. Overall, HCQ in combinatory therapy is being actively used in clinical trials in various type of cancers.

The selective ULK1 inhibitor SBI-0206965 regulates autophagy and cell survival. The combination treatment with SBI-0206965 and mTOR inhibitors promotes the death of tumor cells [168]. ULK-101 is a small molecule inhibitor of ULK1 that inhibits autophagy and the autophagy flux by responding to different stimuli [169]. SAR405 is a low molecular mass kinase inhibitor of PIK3C3 that inhibits autophagy by suppressing the catalytic activity of PIK3C3 [170]. The combination treatment of everolimus and SAR405 exhibits a synergistic anticancer effect via the reduction of cell proliferation in renal cancer cells. SB02024, which is a selective inhibitor of Vps34, is a new highly potent selective inhibitor that improves sensitivity to sunitinib and erlotinib by the inhibition of autophagy by targeting Vps34 [171].

Doxorubicin is a DNA damaging agent, which induces an anticancer effect via the induction of the abnormal function of mitochondria and superoxide production [172]. The inhibition of mitophagy by silencing BNIP3L, a main regulator of mitophagy, restores the sensitivity of doxorubicin in colorectal CSCs [173]. Tanshinone IIA (Tan IIA) is derived from the Chinese medicine Danshen and is used in the treatment of angina, coronary heart disease, hypertension, cerebrovascular diseases, and cancer. In colorectal cancer, treatment with Tan IIA promotes mitochondrial apoptosis by reducing mitophagy by modulating the expression of AMPK and deactivation of Parkin [174]. Mitochondrial division inhibitor 1 (mdivi-1) is a selective inhibitor of mitochondrial division-related protein DRP1 and dynamin I and decreases mitophagy. The inhibition of mitophagy by mdivi-1 enhances silibinin-mediated apoptosis in breast cancer [175].

### 4.2. The Effect of Autophagy Inducers in Anticancer Therapy

The regulation of autophagy plays a protective role in cancer cells against chemotherapy. Therefore, combination therapy with autophagy inhibitors is a good therapeutic strategy for anticancer therapy [176]. In contrast, the excessive induction of autophagy by anticancer drug treatment or autophagy inducers also promotes cancer cell death [177] (Table 2).

Quercetin is a natural flavonol and a multi-kinase inhibitor that restores the sensitivity to ABT-737, and is used as a combination treatment agent with therapeutic effects on leukemic cell lines and B-cells derived from patients via the inhibition of the PI3K/AKT pathway and induction of autophagy [178]. ABT-797 and its derivatives (ABT-263 and ABT-199) have anticancer efficacy in glioblastoma cells via the involvement of autophagic-like cell death by interfering with the interaction with Beclin-1 and Bcl2 [179]. Metformin is used as a synthetic derivative of guanidine against symptoms of diabetes. Additionally, metformin has anticancer effects by inducing autophagy in malignant cells lines and mouse models. Metformin induces the inhibition of cell viability and proliferation via cell cycle arrest and promotes apoptosis by inducing autophagy in endometrial cancer cells [180]. In addition, metformin promotes the TRAIL-mediated apoptosis in TRAIL-resistant lung cancer cells by inducing the autophagic flux via the accumulation of LC3-II and a decrease in p62 levels [181].

Salinomycin (Sal) induces death in several cancer cells via the regulation of various signal pathways, with effects such as the accumulation of dysfunctional mitochondria and induction of ER stress [182]. However, the autophagy inhibitor 3-MA reduces Sal-mediated cell death in melanoma cells via an increase in autophagic markers and reduced formation of autophagosomes. Esomeprazole restores the sensitivity of paclitaxel in non-small cell lung cancer by inhibiting V-ATPase expression and cell proliferation and inducing autophagy, and the treatment with the autophagy inhibitor 3-MA reverses the therapeutic effect of esomeprazole, which enhances paclitaxel-mediated apoptosis [183]. AZD3463 exhibits an anticancer effect as a potential ALK/IGF1R inhibitor and induces apoptosis and autophagy by regulating the PI3K/AKT/mTOR pathway. The co-treatment of AZD3463 and rapamycin increases the efficacy of anticancer therapy via the induction of apoptosis autophagy and reduction of cell proliferation in breast cancer cells [184]. Isoliquiritigenin (ISL) is derived from a flavonoid from *Glycyrrhiza glabra* and shows anticancer effects both in vivo and in vitro. ISL induces the inhibition of cell growth by increasing apoptosis and autophagy via the modulation of the PI3K/AKT/mTOR pathway. Autophagy inhibitor HCQ promotes the therapeutic effect of anticancer therapy against HCC by inducing ISL-mediated apoptosis [185].

The PI3K/mTOR pathway is a promising chemotherapeutic target, which is usually activated in many cancers [186,187]. RAD-001 is known as everolimus and is a derivative of rapamycin. RAD-001 induces sensitivity to paclitaxel-induced apoptosis by activating autophagy via the downregulation of AKT/mTOR phosphorylation and accumulation of LC3 in endometrial cancer and HEC01A cells [188]. Rapamycin activates the expression of Beclin-1 in a dose-dependent manner in pancreatic carcinoma PC-2 cells, and induces autophagic vacuoles, leading to the inhibition of proliferation and induction of apoptosis [189]. In a phase I clinical trial, everolimus was tested in combination with autophagic flux inhibitor HCQ in women, and was found to influence lymphangioleiomyomatosis (NCT01687179). Rapamune (commercial name; rapamycin) has been tested in combination with HCQ in patients with advanced cancer (NCT01266057).

## 5. Conclusions

Autophagy modulates the delicate intracellular processes responding to stressful conditions, such as nutrient deprivation, damaged organelles, and anticancer therapy. Autophagy plays a bipolar role in cell survival and death in cancer initiation and development. Studies with autophagy-defected mouse models have demonstrated that basal levels of autophagy can have a suppressive effect on tumor formation, development, and CSCs. However, the induction of autophagy plays a protective role in cancer progression in many cancers. In addition, autophagy aids in fulfilling the metabolic demand of cancer cells to maintain cancer growth. However, autophagy may also inhibit tumor growth, and the initiation and development of tumors, and CSCs. The different properties of autophagy have been used for devising different potent therapies against different cancers. Additional preclinical studies in a variety of biological fields are essential for a better understanding of the dual role of autophagy in cancer and working to clinical trials for cancer therapy using autophagy regulator. The targeting of autophagy is necessary to develop effective therapeutic strategies for anticancer therapy. In addition, clinical trials testing the efficacy of anticancer therapies utilizing different combinations of autophagy modulators and anticancer drugs are needed (Table 3).

## Figures and Tables

**Table 1:** The role of autophagy inhibitors in cancer therapy.

Compound	Combination Treatment	Cancer Type	Experimental Model	Function	Reference
ATG5 siRNA Beclin-1 siRNA	Cisplatin	Lung cancer (A549)	In vitro	Inhibition of autophagy Restore the sensitivity of cisplatin Enhancement of cisplatin-mediated apoptosis Upregulation of caspase activity Reduction of cell viability	[155]
3-MA		Colorectal cancer (HCT116)	In vitro	Promotion of hypoxia-mediated apoptosis	[9]
Enzalutamide	Chloroquine 3-MA Bafilomycin A1	Bladder cancer (J82, T24, and UMUC3)	In vitro /In vivo	Restores the sensitivity against ENZ Reduction of autophagy and tumor growth Induction of apoptosis	[156]
Atorvastatin	3-MA Bafilomycin A1	Cervical cancer (SiHa and Caski)	In vitro /In vivo	Enhancement of ATO-mediated apoptosis Reduction of autophagy	[157]
Reactivation of p53 and induction of tumor cell apoptosis (RITA)	3-MA	Head and neck cancer (AMC-HN2-10)	In vitro /In vivo	Promotion of therapeutic effects of cisplatin resistance or RITA-resistant cancer Inhibition of autophagy Induction of apoptosis	[158]
Bafilomycin A1		Pediatric B-cell acute lymphoblastic leukemia (RS4;11, NB4, HL-60, K562 and BV173)	In vitro /In vivo	Therapeutic effect at low concentrations Inhibition of autophagy Targeting mitochondria Induction of apoptosis	[160]
Cisplatin	Bafilomycin A1 chloroquine	Bladder cancer (5637 and T25)	In vitro	Enhancement of the therapeutic effect to cisplatin Inhibition of autophagy	[161]
Bafilomycin A1	5-FU	Gastric cancer (SGC-7901)	In vitro	Inhibition of cell viability, colony formation, invasion, and migration Enhancement of apoptosis Suppression of autophagy	[162]
Chloroquine and hydroxychloroquine		Bladder cancer (RT4, 5637, T24, PC3, and MCF-7)	In vitro	Inhibition of autophagy Induction of apoptosis	[163]
Recombinant *Bacillus caldovelox* arginase mutant	Chloroquine	Cervical cancer (Hela, ME-180, C-33A and SiHa)	In vitro	Reduction of tumor growth Increased apoptosis and cell cycle arrest Reduction of autophagy	[164]
SBI-0206965	mTOR inhibitors	Prostate cancer Lung cancer glioblastoma (HEK-293T, U87MG, PC3 and A549)	In vitro	Inhibition of autophagy Reduction of cell survival Promotion of cell death	[169]
SAR405	Everolimus	Renal cancer	In vitro	Inhibition of autophagy Suppression of catalytic activity of PI3KC3 Reduction of cell proliferation	[170]
SB02024	Sunitinib Erlotinib	Breast cancer (HOS and MDA-MB-231)	In vitro /In vivo	Inhibition of autophagy Improvement of sensitivity to Sunitinib and Erlotinib	[171]
Doxorubicin	BNIP3L	Colorectal cancer (HCT8)	In vitro	Inhibition of mitophagy Restoration of the sensitivity of doxorubicin	[173]
Tanshinone IIA	3-MA	Colorectal cancer (SW837 and SW480)	In vitro	Reduction of mitophagy Promotion of mitochondrial apoptosis Decrease of AMPK and Parkin	[174]
Mitochondrial division inhibitor 1	Silibinin	Breast cancer (MCF7 and MDA-MB-231)	In vitro	Inhibition of DRP1 and Dynamin I Decrease of mitophagy Enhancement of silibinin-induced apoptosis	[175]

**Table 2:** The role of autophagy promoters in cancer therapy.

Compound	Combination Treatment	Cancer Type	Experimental Model	Function	Reference
Quercetin	ABT-737 ABT-263	Leukemic cell lines B-cells (HG3)	In vitro	Inhibition of the PI3K/AKT pathway Induction of autophagy Restoration of the sensitivity to ABT-737	[178]
ABT-737 ABT-263 ABT-199		Glioblastoma cells	In vitro	Induction of autophagic cell death Interruption of the interaction with Beclin-1 and Bcl2	[179]
Metformin	3-MA Chloroquine	Endometrial cancer cells (Ishikawa cells)	In vitro	Inhibition of cell viability and proliferation Increased cell cycle arrest and apoptosis Enhancement of autophagy	[180]
		TRAIL-resistant lung cancer (A549, Calu-3 and HCC-15)	In vitro	Promotion of autophagic flux Accumulation of LC3-II Reduction of p62	[181]
Salinomycin		Melanoma cells (M7, M8, M21, M29, SK-MEL-1, SK-MEL-12 and A375)	In vitro /In vivo	Induction of cell death Accumulation of abnormal mitochondria Increased ER stress	[182]
Esomeprazole	Paclitaxel	Non-small cell lung cancer (A549)	In vitro	Restoration of the sensitivity to paclitaxel Inhibition of V-ATPase and cell proliferation Enhancement of autophagy	[183]
AZD3463	Rapamycin	Breast cancer (MCF7)	In vitro	AZD3463: ALK/IGF1R inhibitor Promotion of apoptosis and autophagy Reduction of cell proliferation	[184]
Isoliquiritigenin		Hepatocellular carcinoma (MHCC97-H, LO_2_ and SMMC7721)	In vitro /In vivo	Inhibition of cell growth Enhancement of apoptosis and autophagy Modulation of the PI3K/AKT/mTOR pathway	[185]
RAD-001	Paclitaxel	Endometrial cancer cells (Ishikawa and HEC-1A)	In vitro	Induction of sensitivity to paclitaxel Promotion of apoptosis and autophagy Downregulation of AKT/mTOR Accumulation of LC3	[188]
Rapamycin		Pancreatic carcinoma (PC-2)	In vitro	Activation of Beclin-1 Induction of autophagic vacuoles Inhibition of proliferation and induction of apoptosis	[189]
Everolimus	Hydroxychloroquine	Lymphangioleiomyomatosis	Phase I /Complete	Investigation of the effect on the regulation of autophagy in lymphangioleiomyomatosis	NCT01687179
Rapamune	Hydroxychloroquine	Advanced cancer	Phase I /Active	Investigation of the effect on the regulation of autophagy in advanced cancer	NCT01266057

**Table 3:** Clinical trials using autophagy modulators in cancer therapy.

NCT Number	Title	Status	Cancer Type	Drugs	Phase
NCT03037437	Sorafenib Induced Autophagy Using Hydroxychloroquine in Hepatocellular Cancer	Recruiting	Hepatocellular cancer	Sorafenib Hydroxychloroquine	Phase II
NCT01649947	Modulation of Autophagy in Patients With Advanced/Recurrent Non-small Cell Lung Cancer	Complete	Non-small cell lung cancer	Paclitaxel Carboplatin Hydroxychloroquine Bevacizumab	Phase II
NCT04214418	Study of Combination Therapy With the MEK Inhibitor, Cobimetinib, Immune Checkpoint Blockade, Atezolizumab, and the AUTOphagy Inhibitor, Hydroxychloroquine in KRAS-mutated Advanced Malignancies	Recruiting	Gastrointestinal cancer	Cobimetinib Hydroxychloroquine Atezolizumab	Phase I/II
NCT04333914	Prospective Study in Patients With Advanced or Metastatic Cancer and SARS-CoV-2 Infection	Recruiting	Advanced or Metastatic Hematological or Solid Tumor	Autophagy inhibitor (GNS651) Avdoralimab Monalizumab	Phase II
NCT01206530	FOLFOX/Bevacizumab/Hydroxychloroquine (HCQ) in Colorectal Cancer	Complete	Rectal and colon cancer	Hydroxychloroquine Oxaliplatin Leucovorin	Phase I/II
NCT03774472	Hydroxychloroquine, Palbociclib, and Letrozole Before Surgery in Treating Participants With Estrogen Receptor Positive, HER2 Negative Breast Cancer	Recruiting	Breast cancer	Hydroxychloroquine Letrozole Palbociclib	Phase I/II
NCT02316340	Vorinostat Plus Hydroxychloroquine Versus Regorafenib in Colorectal Cancer	Complete	Colorectal cancer	Voriostat Hydroxychloroquine Regorafenib	Phase II
NCT04132505	Binimetinib and Hydroxychloroquine in Treating Patients With KRAS Mutant Metastatic Pancreatic Cancer	Recruiting	Pancreatic cancer	Binimetinib Hydroxychloroquine	Phase I
NCT04524702	Paricalcitol and Hydroxychloroquine in Combination With Gemcitabine and Nab-Paclitaxel for the Treatment of Advanced or Metastatic Pancreatic Cancer	Recruiting	Pancreatic cancer	Cemcitabine Hydroxychloroquine Nab-paclitaxel Paricaitol	Phase II
NCT03377179	A Study of ABC294640 (Yeliva ^®^) Alone and in Combination With Hydroxychloroquine Sulfate in Treatment of Patients With Advanced Cholangiocarcinoma	Recruiting	Cholangiocarcinoma	ABC294640 Hydroxychloroquine	Phase II
NCT04163107	Combined Carfilzomib and Hydroxychloroquine in Patients With Relapsed/Refractory Multiple Myeloma	Recruiting	Multiple Myeloma	Hydroxychloroquine Carfizomib Dexamethasone	Phase I
NCT03598595	Gemcitabine, Docetaxel, and Hydroxychloroquine in Treating Participants With Recurrent or Refractory Osteosarcoma	Recruiting	Osteosarcoma	Docetaxel Gemcitabine Hydroxychloroquine	Phase I/II

## References

[1] Onorati A.V., Dyczynski M., Ojha R., Amaravadi R.K. (2018). Targeting autophagy in cancer. Cancer.

[2] Parzych K.R., Klionsky D.J. (2014). An Overview of Autophagy: Morphology, Mechanism, and Regulation. Antioxid. Redox Signal..

[3] Huang T., Song X., Yang Y., Wan X., Alvarez A.A., Sastry N., Feng H., Hu B., Cheng S.-Y. (2018). Autophagy and Hallmarks of Cancer. Crit. Rev. Oncog..

[4] Glick D., Barth S., MacLeod K.F. (2010). Autophagy: Cellular and molecular mechanisms. J. Pathol..

[5] Guo F., Liu X., Cai H., Le W. (2017). Autophagy in neurodegenerative diseases: Pathogenesis and therapy. Brain Pathol..

[6] Mialet-Perez J., Vindis C. (2017). Autophagy in health and disease: Focus on the cardiovascular system. Essays Biochem..

[7] Sarparanta J., García-Macia M., Singh R. (2017). Autophagy and Mitochondria in Obesity and Type 2 Diabetes. Curr. Diabetes Rev..

[8] Yun C.W., Lee S.H. (2018). The Roles of Autophagy in Cancer. Int. J. Mol. Sci..

[9] Dong Y., Wu Y., Zhao G.L., Ye Z.Y., Xing C.G., Yang X.D. (2019). Inhibition of autophagy by 3-MA promotes hypox-ia-induced apoptosis in human colorectal cancer cells. Eur. Rev. Med. Pharmacol. Sci..

[10] Endo S., Nakata K., Sagara A., Koikawa K., Ando Y., Kibe S., Takesue S., Nakayama H., Abe T., Okumura T. (2017). Autophagy inhibition enhances antiproliferative effect of salinomycin in pancreatic cancer cells. Pancreatology.

[11] Asl E.R., Mansori B., Mohammadi A., Najafi S., Pouya F.D., Rami Y. (2020). Interaction between DNA damage response and autophagy in colorectal cancer. Gene.

[12] Anderson C.M., MacLeod K.F. (2019). Autophagy and cancer cell metabolism. Int. Rev. Cell Mol. Biol..

[13] Xiong X., Lu B., Tian Q., Zhang H., Wu M., Guo H., Zhang Q., Li X., Zhou T., Wang Y. (2018). Inhibition of autophagy enhances cinobufagin-induced apoptosis in gastric cancer. Oncol. Rep..

[14] White E., Mehnert J.M., Chan C.S. (2015). Autophagy, Metabolism, and Cancer. Clin. Cancer Res..

[15] Yorimitsu T., Klionsky D.J. (2005). Autophagy: Molecular machinery for self-eating. Cell Death Differ..

[16] Oku M., Sakai Y. (2018). Three Distinct Types of Microautophagy Based on Membrane Dynamics and Molecular Machineries. BioEssays.

[17] Kaushik S., Cuervo A.M. (2018). The coming of age of chaperone-mediated autophagy. Nat. Rev. Mol. Cell Biol..

[18] Shibutani S.T., Yoshimori T. (2014). A current perspective of autophagosome biogenesis. Cell Res..

[19] Gui D., Cui Z., Zhang L., Yu C., Yao D., Xu M., Chen M., Wu P.L., Liangxing W., Wang L. (2017). Salidroside attenuates hypoxia-induced pulmonary arterial smooth muscle cell proliferation and apoptosis resistance by upregulating autophagy through the AMPK-mTOR-ULK1 pathway. BMC Pulm. Med..

[20] He C., Klionsky D.J. (2009). Regulation mechanisms and signaling pathways of autophagy. Annu. Rev. Genet..

[21] Levine B., Klionsky D.J. (2004). Development by Self-Digestion. Dev. Cell.

[22] Matsunaga K., Saitoh T., Tabata K., Omori H., Satoh T., Kurotori N., Maejima I., Shirahama-Noda K., Ichimura T., Isobe T. (2009). Two Beclin 1-binding proteins, Atg14L and Rubicon, reciprocally regulate autophagy at different stages. Nat. Cell Biol..

[23] Walczak M., Martens S. (2013). Dissecting the role of the Atg12–Atg5-Atg16 complex during autophagosome formation. Autophagy.

[24] Dooley H.C., Razi M., Polson H.E., Girardin S.E., Wilson M.I., Tooze S.A. (2014). WIPI2 Links LC3 Conjugation with PI3P, Autophagosome Formation, and Pathogen Clearance by Recruiting Atg12–5-16L1. Mol. Cell.

[25] Schaaf M.B.E., Keulers T.G., Vooijs M.A., Rouschop K.M.A. (2016). LC3/GABARAP family proteins: Autophagy-(un)related functions. FASEB J..

[26] Reggiori F., Ungermann C. (2017). Autophagosome Maturation and Fusion. J. Mol. Biol..

[27] Ni H.-M., Williams J.A., Ding W.-X. (2015). Mitochondrial dynamics and mitochondrial quality control. Redox Biol..

[28] Pickles S., Vigié P., Youle R.J. (2018). Mitophagy and Quality Control Mechanisms in Mitochondrial Maintenance. Curr. Biol..

[29] Palikaras K., Lionaki E., Tavernarakis N. (2018). Mechanisms of mitophagy in cellular homeostasis, physiology and pathology. Nat. Cell Biol..

[30] Jin S.M., Youle R.J. (2012). PINK1- and Parkin-mediated mitophagy at a glance. J. Cell Sci..

[31] Ordureau A., Heo J.-M., Duda D.M., Paulo J.A., Olszewski J.L., Yanishevski D., Rinehart J., Schulman B.A., Harper J.W. (2015). Defining roles of PARKIN and ubiquitin phosphorylation by PINK1 in mitochondrial quality control using a ubiquitin replacement strategy. Proc. Natl. Acad. Sci. USA.

[32] Durcan T.M., Fon E.A. (2015). The three ‘P’s of mitophagy: PARKIN, PINK1, and post-translational modifications. Genes Dev..

[33] Von Stockum S., Marchesan E., Ziviani E. (2018). Mitochondrial quality control beyond PINK1/Parkin. Oncotarget.

[34] Ding W.-X., Yin X.-M. (2012). Mitophagy: Mechanisms, pathophysiological roles, and analysis. Biol. Chem..

[35] Zhang J., Ney P.A. (2009). Role of BNIP3 and NIX in cell death, autophagy, and mitophagy. Cell Death Differ..

[36] Yoo S.-M., Jung Y.-K. (2018). A Molecular Approach to Mitophagy and Mitochondrial Dynamics. Mol. Cells.

[37] Kirchner P., Bourdenx M., Madrigal-Matute J., Tiano S., Diaz A., Bartholdy B.A., Will B., Cuervo A.M. (2019). Proteome-wide analysis of chaperone-mediated autophagy targeting motifs. PLoS Biol..

[38] Bandyopadhyay U., Kaushik S., Varticovski L., Cuervo A.M. (2008). The Chaperone-Mediated Autophagy Receptor Organizes in Dynamic Protein Complexes at the Lysosomal Membrane. Mol. Cell. Biol..

[39] Bandyopadhyay U., Sridhar S., Kaushik S., Kiffin R., Cuervo A.M. (2010). Identification of Regulators of Chaperone-Mediated Autophagy. Mol. Cell.

[40] Cuervo A.M., Dice J.F. (2000). Regulation of Lamp2a Levels in the Lysosomal Membrane. Traffic.

[41] Saha S., Panigrahi D.P., Patil S., Bhutia S.K. (2018). Autophagy in health and disease: A comprehensive review. Biomed. Pharmacother..

[42] Cordani M., Butera G., Pacchiana R., Donadelli M. (2017). Molecular interplay between mutant p53 proteins and autophagy in cancer cells. Biochim. Biophys. Acta (BBA) Bioenerg..

[43] Liao J.-K., Zhou B., Zhuang X.-M., Zhuang P.-L., Zhang D.-M., Chen W.-L. (2018). Cancer-associated fibroblasts confer cisplatin resistance of tongue cancer via autophagy activation. Biomed. Pharmacother..

[44] Mainz L., Rosenfeldt M.T. (2017). Autophagy and cancer—insights from mouse models. FEBS J..

[45] Nazio F., Bordi M., Cianfanelli V., Locatelli F., Cecconi F. (2019). Autophagy and cancer stem cells: Molecular mechanisms and therapeutic applications. Cell Death Differ..

[46] Devettere R.J. (1992). Slippery slopes and moral reasoning. J. Clin. Ethic..

[47] Takahashi Y., Coppola D., Matsushita N., Cualing H.D., Sun M., Sato Y., Liang C., Jung J.U., Cheng J.Q., Mulé J.J. (2007). Bif-1 interacts with Beclin 1 through UVRAG and regulates autophagy and tumorigenesis. Nat. Cell Biol..

[48] Toton E., Lisiak N., Sawicka P., Rybczynska M. (2014). Beclin-1 and its role as a target for anticancer therapy. J. Physiol. Pharmacol. Off. J. Pol. Physiol. Soc..

[49] Tao H., Chen F., Liu H., Hu Y., Wang Y., Yanling H. (2017). Wnt/β-catenin signaling pathway activation reverses gemcitabine resistance by attenuating Beclin1-mediated autophagy in the MG63 human osteosarcoma cell line. Mol. Med. Rep..

[50] Vega-Rubín-De-Celis S., Zou Z., Fernández Á.F., Ci B., Kim M., Xiao G., Xie Y., Levine B. (2018). Increased autophagy blocks HER2-mediated breast tumorigenesis. Proc. Natl. Acad. Sci. USA.

[51] Peng Y., Miao H., Wu S., Yang W., Zhang Y., Xie G., Xie X., Li J., Shi C., Ye L. (2016). ABHD5 interacts with BECN1 to regulate autophagy and tumorigenesis of colon cancer independent of PNPLA2. Autophagy.

[52] Lee J.W., Jeong E.G., Lee S.H., Yoo N.J., Lee S.H. (2007). Somatic mutations of BECN1, an autophagy-related gene, in human cancers. APMIS.

[53] Laddha S.V., Ganesan S., Chan C.S., White E. (2014). Mutational Landscape of the Essential Autophagy Gene BECN1 in Human Cancers. Mol. Cancer Res..

[54] Qu X., Yu J., Bhagat G., Furuya N., Hibshoosh H., Troxel A., Rosen J., Eskelinen E.-L., Mizushima N., Ohsumi Y. (2003). Promotion of tumorigenesis by heterozygous disruption of the beclin 1 autophagy gene. J. Clin. Investig..

[55] Takamura A., Komatsu M., Hara T., Sakamoto A., Kishi C., Waguri S., Eishi Y., Hino O., Tanaka K., Mizushima N. (2011). Autophagy-deficient mice develop multiple liver tumors. Genes Dev..

[56] Mathew R., Karp C.M., Beaudoin B., Vuong N., Chen G., Chen H.-Y., Bray K., Reddy A., Bhanot G., Gelinas C. (2009). Autophagy Suppresses Tumorigenesis through Elimination of p62. Cell.

[57] Saito T., Ichimura Y., Taguchi K., Suzuki T., Mizushima T., Takagi T.M.K., Hirose Y., Nagahashi M., Iso T., Fukutomi T. (2016). p62/Sqstm1 promotes malignancy of HCV-positive hepatocellular carcinoma through Nrf2-dependent metabolic reprogramming. Nat. Commun..

[58] Moscat J., Karin M., Diaz-Meco M.T. (2016). p62 in Cancer: Signaling Adaptor Beyond Autophagy. Cell.

[59] Wu Q., Xiang M., Wang K., Chen Z., Long L., Tao Y., Liang Y., Yan Y., Xiao Z., Qiu S. (2020). Overexpression of p62 Induces Autophagy and Promotes Proliferation, Migration and Invasion of Nasopharyngeal Carcinoma Cells through Promoting ERK Signaling Pathway. Curr. Cancer Drug Targets.

[60] Hwang S.K., Jeong Y.J., Chang Y.C. (2020). PDCD4 inhibits lung tumorigenesis by the suppressing p62-Nrf2 signaling pathway and upregulating Keap1 expression. Am. J. Cancer Res..

[61] Vara-Perez M., Felipe-Abrio B., Agostinis P. (2019). Mitophagy in Cancer: A Tale of Adaptation. Cells.

[62] Chang J.Y., Yi H.-S., Kim H.-W., Shong M. (2017). Dysregulation of mitophagy in carcinogenesis and tumor progression. Biochim. Biophys. Acta (BBA) Bioenerg..

[63] Wang Y., Liu H.-H., Cao Y.-T., Zhang L.-L., Huang F., Yi C. (2020). The Role of Mitochondrial Dynamics and Mitophagy in Carcinogenesis, Metastasis and Therapy. Front. Cell Dev. Biol..

[64] Panigrahi D.P., Praharaj P.P., Bhol C.S., Mahapatra K.K., Patra S., Behera B.P., Mishra S.R., Bhutia S.K. (2020). The emerging, multifaceted role of mitophagy in cancer and cancer therapeutics. Semin. Cancer Biol..

[65] Barazzuol L., Giamogante F., Brini M., Calì T. (2020). PINK1/Parkin Mediated Mitophagy, Ca2+ Signalling, and ER–Mitochondria Contacts in Parkinson’s Disease. Int. J. Mol. Sci..

[66] Fujiwara M., Marusawa H., Wang H.-Q., Iwai A., Ikeuchi K., Imai Y., Kataoka A., Nukina N., Takahashi R., Chiba T. (2008). Parkin as a tumor suppressor gene for hepatocellular carcinoma. Oncogene.

[67] Zhang X., Lin C., Song J., Chen H., Chen X., Ren L., Zhou Z., Pan J., Yang Z., Bao W. (2019). Parkin facilitates proteasome inhibitor-induced apoptosis via suppression of NF-κB activity in hepatocellular carcinoma. Cell Death Dis..

[68] Lu T.-L., Huang G.-J., Wang H.-J., Chen J.-L., Hsu H.-P. (2010). Hispolon promotes MDM2 downregulation through chaperone-mediated autophagy. Biochem. Biophys. Res. Commun..

[69] Bonhoure A., Vallentin A., Martin M., Senff-Ribeiro A., Amson R., Telerman A., Vidal M. (2017). Acetylation of translationally controlled tumor protein promotes its degradation through chaperone-mediated autophagy. Eur. J. Cell Biol..

[70] Schneider J.L., Villarroya J., Diaz-Carretero A., Patel B., Urbanska A.M., Thi M.M., Villarroya F., Santambrogio L., Cuervo A.M. (2015). Loss of hepatic chaperone-mediated autophagy accelerates proteostasis failure in aging. Aging Cell.

[71] Kaushik S., Cuervo A.M. (2015). Degradation of lipid droplet-associated proteins by chaperone-mediated autophagy facilitates lipolysis. Nat. Cell Biol..

[72] Chatterjee S., Seth R.K., Kumar A., Kadiiska M.B., Michelotti G., Diehl A.M., Chatterjee S. (2013). Purinergic receptor X7 is a key modulator of metabolic oxidative stress-mediated autophagy and inflammation in experimental nonalcoholic steatohepatitis. Am. J. Physiol. Liver Physiol..

[73] Rodriguez-Navarro J.A., Kaushik S., Koga H., Dall’Armi C., Shui G., Wenk M.R., Di Paolo G., Cuervo A.M. (2012). Inhibitory effect of dietary lipids on chaperone-mediated autophagy. Proc. Natl. Acad. Sci. USA.

[74] Cai Y., Jogasuria A., Yin H., Xu M.-J., Hu X., Wang J., Kim C., Wu J., Lee K., Gao B. (2016). The Detrimental Role Played by Lipocalin-2 in Alcoholic Fatty Liver in Mice. Am. J. Pathol..

[75] Xiongshan S., Deng Y., Pan X., Li P., Lai W., Luo H., Huang P., Guan X., Deng Y., Yan J. (2017). Downregulation of ATG5-dependent macroautophagy by chaperone-mediated autophagy promotes breast cancer cell metastasis. Sci. Rep..

[76] Dillekås H., Rogers M.S., Straume O. (2019). Are 90% of deaths from cancer caused by metastases?. Cancer Med..

[77] Saad A.M., Gad M.M., Al-Husseini M.J., AlKhayat M.A., Rachid A., AlFaar A.S., Hamoda H.M. (2019). Suicidal death within a year of a cancer diagnosis: A population-based study. Cancer.

[78] Kenific C.M., Thorburn A., Debnath J. (2010). Autophagy and metastasis: Another double-edged sword. Curr. Opin. Cell Biol..

[79] Qiang L., Zhao B., Ming M., Wang N., He T.-C., Hwang S., Thorburn A., He Y.-Y. (2014). Regulation of cell proliferation and migration by p62 through stabilization of Twist1. Proc. Natl. Acad. Sci. USA.

[80] Lisanti M.P., Martinez-Outschoorn U.E., Chiavarina B., Pavlides S., Whitaker-Menezes D., Tsirigos A., Witkiewicz A.K., Lin Z., Balliet R.M., Howell A. (2010). Understanding the "lethal" drivers of tumor-stroma co-evolution. Cancer Biol. Ther..

[81] Mowers E.E., Sharifi M.N., MacLeod K.F. (2018). Functions of autophagy in the tumor microenvironment and cancer metastasis. FEBS J..

[82] DeNardo D.G., Barreto J.B., Andreu P., Vasquez L., Tawfik D., Kolhatkar N., Coussens L.M. (2009). CD4+ T Cells Regulate Pulmonary Metastasis of Mammary Carcinomas by Enhancing Protumor Properties of Macrophages. Cancer Cell.

[83] Bingle L., Brown N.J., Lewis C.E. (2002). The role of tumour-associated macrophages in tumour progression: Implications for new anticancer therapies. J. Pathol..

[84] Xia H., Li S., Li X., Wang W., Bian Y., Wei S., Grove S., Wang W., Vatan L., Liu J.R. (2020). Autophagic adaptation to oxidative stress alters peritoneal residential macrophage survival and ovarian cancer metastasis. JCI Insight.

[85] Sharifi M.N., Mowers E.E., Drake L.E., Collier C., Chen H., Zamora M., Mui S., MacLeod K.F. (2016). Autophagy Promotes Focal Adhesion Disassembly and Cell Motility of Metastatic Tumor Cells through the Direct Interaction of Paxillin with LC3. Cell Rep..

[86] Guo W., Giancotti F.G. (2004). Integrin signalling during tumour progression. Nat. Rev. Mol. Cell Biol..

[87] Guadamillas M.C., Cerezo A., Del Pozo M.Á. (2011). Overcoming anoikis—Pathways to anchorage-independent growth in cancer. J. Cell Sci..

[88] Long X.H., Zhou Y.F., Lan M., Huang S.H., Liu Z.L., Shu Y. (2019). Valosin-containing protein promotes metastasis of osteosarcoma through autophagy induction and anoikis inhibition via the ERK/NF-κβ/beclin-1 signaling pathway. Oncol. Lett..

[89] Schoenherr C., Byron A., Sandilands E., Paliashvili K., Baillie G.S., Garcia-Munoz A., Valacca C., Cecconi F., Serrels B., Frame M.C. (2017). Ambra1 spatially regulates Src activity and Src/FAK-mediated cancer cell invasion via trafficking networks. eLife.

[90] Li J., Yang B., Zhou Q., Wu Y., Shang D., Guo Y., Song Z., Zheng Q., Xiong J. (2013). Autophagy promotes hepatocellular carcinoma cell invasion through activation of epithelial–mesenchymal transition. Carcinogenesis.

[91] Fan Q., Yang L., Zhang X., Ma Y., Li Y., Dong L., Zong Z., Hua X., Su D., Li H. (2018). Autophagy promotes metastasis and glycolysis by upregulating MCT1 expression and Wnt/β-catenin signaling pathway activation in hepatocellular carcinoma cells. J. Exp. Clin. Cancer Res..

[92] Zhai H., Fesler A., Ba Y., Wu S., Ju J. (2015). Inhibition of colorectal cancer stem cell survival and invasive potential by hsa-miR-140-5p mediated suppression of Smad2 and autophagy. Oncotarget.

[93] Zada S., Hwang J.S., Ahmed M., Lai T., Pham T.M., Kim D.R. (2019). Control of the Epithelial-to-Mesenchymal Transition and Cancer Metastasis by Autophagy-Dependent SNAI1 Degradation. Cells.

[94] Liberti M.V., Locasale J.W. (2016). The Warburg Effect: How Does it Benefit Cancer Cells?. Trends Biochem. Sci..

[95] Bernardini J.P., Lazarou M., Dewson G. (2017). Parkin and mitophagy in cancer. Oncogene.

[96] Zhang C., Lin M., Wu R., Wang X., Yang B., Levine A.J., Hu W., Feng Z. (2011). Parkin, a p53 target gene, mediates the role of p53 in glucose metabolism and the Warburg effect. Proc. Natl. Acad. Sci. USA.

[97] Liu J., Zhang C., Gatza M.L., Xia D., Gao J., White E., Haffty B.G., Hu W., Feng Z., Zhao Y. (2017). Parkin targets HIF-1α for ubiquitination and degradation to inhibit breast tumor progression. Nat. Commun..

[98] Lee Y.S., Jung Y.Y., Park M.H., Yeo I.J., Im H.S., Nam K.T., Kim H.D., Kang S.K., Song J.K., Kim Y.R. (2018). Deficiency of parkin suppresses melanoma tumor development and metastasis through inhibition of MFN2 ubiquitination. Cancer Lett..

[99] Gustafsson Å.B. (2011). Bnip3 as a Dual Regulator of Mitochondrial Turnover and Cell Death in the Myocardium. Pediatr. Cardiol..

[100] Chourasia A.H., Tracy K., MacLeod K.F., Frankenberger C.A., Boland M.L., Sharifi M.N., Drake L.E., Sachleben J.R., Asara J.M., Locasale J.W. (2015). Mitophagy defects arising from BNip3 loss promote mammary tumor progression to metastasis. EMBO Rep..

[101] Shi C., Cai Y., Li Y., Li Y., Hu N., Ma S., Hu S., Zhu P., Wang W., Zhou H. (2018). Yap promotes hepatocellular carcinoma metastasis and mobilization via governing cofilin/F-actin/lamellipodium axis by regulation of JNK/Bnip3/SERCA/CaMKII pathways. Redox Biol..

[102] Kon M., Kiffin R., Koga H., Chapochnick J., Macian F., Varticovski L., Cuervo A.M. (2011). Chaperone-Mediated Autophagy Is Required for Tumor Growth. Sci. Transl. Med..

[103] Zhang Y., Xu Y.-Y., Yao C.-B., Li J.-T., Zhao X.-N., Yang H.-B., Zhang M., Yin M., Chen J., Lei Q.-Y. (2017). Acetylation targets HSD17B4 for degradation via the CMA pathway in response to estrone. Autophagy.

[104] Li Y.-J., Lei Y.-H., Yao N., Wang C.-R., Hu N., Ye W.-C., Zhang D.-M., Chen Z. (2017). Autophagy and multidrug resistance in cancer. Chin. J. Cancer.

[105] Kumar A., Singh U.K., Chaudhary A. (2015). Targeting autophagy to overcome drug resistance in cancer therapy. Future Med. Chem..

[106] Wang F., Xia X., Yang C., Shen J., Mai J., Kim H.-C., Kirui D., Kang Y., Fleming J.B., Koay E.J. (2018). SMAD4Gene Mutation Renders Pancreatic Cancer Resistance to Radiotherapy through Promotion of Autophagy. Clin. Cancer Res..

[107] Taylor M.A., Das B.C., Ray S.K. (2018). Targeting autophagy for combating chemoresistance and radioresistance in glioblastoma. Apoptosis.

[108] Hao C., Liu G., Tian G. (2019). Autophagy inhibition of cancer stem cells promotes the efficacy of cisplatin against non-small cell lung carcinoma. Ther. Adv. Respir. Dis..

[109] Yeo S.K., Guan J.-L. (2016). Hierarchical heterogeneity in mammary tumors and its regulation by autophagy. Autophagy.

[110] Eapen V.V., Waterman D.P., Chuartzman S.G., Loewith R.J., Schuldiner M., Denic V., Klionsky D.J., Haber J.E., Bernard A., Schiffmann N. (2017). A pathway of targeted autophagy is induced by DNA damage in budding yeast. Proc. Natl. Acad. Sci. USA.

[111] Weisberg E., Nonami A., Griffin J.D. (2014). Combination therapy with nilotinib for drug-sensitive and drug-resistant BCR-ABL-positive leukemia and other malignancies. Arch. Toxicol..

[112] Cao L., Yang L., Zhang H., Wang Z., Yu Y., Xie M., Zhao M., Liu L. (2011). S100A8-targeting siRNA enhances arsenic trioxide-induced myeloid leukemia cell death by down-regulating autophagy. Int. J. Mol. Med..

[113] Xiao X., Wang W., Li Y., Yang D., Li X., Shen C., Liu Y., Ke X., Guo S., Guo Z. (2018). HSP90AA1-mediated autophagy promotes drug resistance in osteosarcoma. J. Exp. Clin. Cancer Res..

[114] Shang J., Chen W.-M., Liu S., Wang Z.-H., Wei T.-N., Chen Z.-Z., Wu W.-B. (2019). CircPAN3 contributes to drug resistance in acute myeloid leukemia through regulation of autophagy. Leuk. Res..

[115] Santiago-O’Farrill J.M., Weroha S.J., Hou X., Oberg A.L., Bs E.P.H., Ms M.J.M., Pang L., Rask P., Amaravadi R.K., Becker S.E. (2019). Poly(adenosine diphosphate ribose) polymerase inhibitors induce autophagy-mediated drug resistance in ovarian cancer cells, xenografts, and patient-derived xenograft models. Cancer.

[116] Datta S., Choudhury D., Das A., Das Mukherjee D., Dasgupta M., Bandopadhyay S., Chakrabarti G. (2019). Autophagy inhibition with chloroquine reverts paclitaxel resistance and attenuates metastatic potential in human nonsmall lung adenocarcinoma A549 cells via ROS mediated modulation of β-catenin pathway. Apoptosis.

[117] Jiang K., Zhang C., Yu B., Chen B., Liu Z., Hou C., Wang F., Shen H., Chen Z. (2017). Autophagic degradation of FOXO3a represses the expression of PUMA to block cell apoptosis in cisplatin-resistant osteosarcoma cells. Am. J. Cancer Res..

[118] Chen S., Wu J., Jiao K., Wu Q., Ma J., Chen D., Kang J., Zhao G., Shi Y., Fan D. (2018). MicroRNA-495-3p inhibits multidrug resistance by modulating autophagy through GRP78/mTOR axis in gastric cancer. Cell Death Dis..

[119] Du X., Liu B., Luan X., Cui Q., Li L. (2017). miR-30 decreases multidrug resistance in human gastric cancer cells by modulating cell autophagy. Exp. Ther. Med..

[120] Scambia G., Panici P., Baiocchi G., Perrone L., Greggi S., Mancuso S. (1988). CA 15-3 as a tumor marker in gynecological malignancies. Gynecol. Oncol..

[121] Zhang H., Tang J., Li C., Kong J., Wang J., Wu Y., Xu E., Lai M.-D. (2015). MiR-22 regulates 5-FU sensitivity by inhibiting autophagy and promoting apoptosis in colorectal cancer cells. Cancer Lett..

[122] Yu Y., Xiang N., Lin M., Huang J.-W., Zhang J., Cheng B., Ji C. (2019). miR- 26a Sensitizes Melanoma Cells To Dabrafenib Via Targeting HMGB1-Dependent Autophagy Pathways. Drug Des. Dev. Ther..

[123] Yamashita K., Miyata H., Makino T., Masuike Y., Furukawa H., Tanaka K., Miyazaki Y., Takahashi T., Kurokawa Y., Yamasaki M. (2017). High Expression of the Mitophagy-Related Protein Pink1 is Associated with a Poor Response to Chemotherapy and a Poor Prognosis for Patients Treated with Neoadjuvant Chemotherapy for Esophageal Squamous Cell Carcinoma. Ann. Surg. Oncol..

[124] Oun R., Moussa Y.E., Wheate N.J. (2018). Correction: The side effects of platinum-based chemotherapy drugs: A review for chemists. Dalton Trans..

[125] Villa E., Proïcs E., Rubio-Patiño C., Obba S., Zunino B., Bossowski J.P., Rozier R.M., Chiche J., Mondragón L., Riley J.S. (2017). Parkin-Independent Mitophagy Controls Chemotherapeutic Response in Cancer Cells. Cell Rep..

[126] Yao N., Wang C., Chen W.-M., Chen Z., Fu D., Ye W., Zhang D.-M., Hu N., Li Y., Liu M. (2019). Inhibition of PINK1/Parkin-dependent mitophagy sensitizes multidrug-resistant cancer cells to B5G1, a new betulinic acid analog. Cell Death Dis..

[127] Basit F., Van Oppen L.M., Schöckel L., Bossenbroek H.M., Vries S.E.V.E.-D., Hermeling J.C., Grefte S., Kopitz C., Heroult M., Willems P.H. (2017). Mitochondrial complex I inhibition triggers a mitophagy-dependent ROS increase leading to necroptosis and ferroptosis in melanoma cells. Cell Death Dis..

[128] Zhou J., Li G., Zheng Y., Shen H.-M., Hu X., Ming Q.-L., Huang C., Li P., Gao N. (2015). A novel autophagy/mitophagy inhibitor liensinine sensitizes breast cancer cells to chemotherapy through DNM1L-mediated mitochondrial fission. Autophagy.

[129] Lobo N.A., Shimono Y., Qian D., Clarke M.F. (2007). The Biology of Cancer Stem Cells. Annu. Rev. Cell Dev. Biol..

[130] Bonnet D., Dick J.E. (1997). Human acute myeloid leukemia is organized as a hierarchy that originates from a primitive hematopoietic cell. Nat. Med..

[131] Bellodi C., Lidonnici M.R., Hamilton A., Helgason G.V., Soliera A.R., Ronchetti M., Galavotti S., Young K.W., Selmi T., Yacobi R. (2009). Targeting autophagy potentiates tyrosine kinase inhibitor–induced cell death in Philadelphia chromosome–positive cells, including primary CML stem cells. J. Clin. Investig..

[132] Jiang H., Gomez-Manzano C., Aoki H., Alonso M.M., Kondo S., McCormick F., Xu J., Kondo Y., Bekele B.N., Colman H. (2007). Examination of the Therapeutic Potential of Delta-24-RGD in Brain Tumor Stem Cells: Role of Autophagic Cell Death. J. Natl. Cancer Inst..

[133] Zhu H., Wang D., Liu Y., Su Z., Zhang L., Chen F., Zhou Y., Wu Y., Yu M., Zhang Z. (2013). Role of the Hypoxia-inducible factor-1 alpha induced autophagy in the conversion of non-stem pancreatic cancer cells into CD133+ pancreatic cancer stem-like cells. Cancer Cell Int..

[134] Guan J.-L., Simon A.K., Prescott M., Menendez J.A., Liu F., Wang F., Wang C., Wolvetang E.J., Vazquez-Martin A., Zhang J. (2013). Autophagy in stem cells. Autophagy.

[135] O’Brien C.A., Pollett A., Gallinger S., Dick J.E. (2006). A human colon cancer cell capable of initiating tumour growth in immunodeficient mice. Nature.

[136] Ricci-Vitiani L., Lombardi D.G., Pilozzi E., Biffoni M., Todaro M., Peschle C., De Maria R. (2006). Identification and expansion of human colon-cancer-initiating cells. Nature.

[137] Gong C., Bauvy C., Tonelli G., Yue W., Delomenie C., Nicolas V., Zhu Y., Domergue V., Marinesteban V., Tharinger H. (2012). Beclin 1 and autophagy are required for the tumorigenicity of breast cancer stem-like/progenitor cells. Oncogene.

[138] Chaterjee M., Van Golen K.L. (2011). Breast Cancer Stem Cells Survive Periods of Farnesyl-Transferase Inhibitor-Induced Dormancy by Undergoing Autophagy. Bone Marrow Res..

[139] Hermann P.C., Huber S.L., Herrler T., Aicher A., Ellwart J.W., Guba M., Bruns C.J., Heeschen C. (2007). Distinct Populations of Cancer Stem Cells Determine Tumor Growth and Metastatic Activity in Human Pancreatic Cancer. Cell Stem Cell.

[140] Li C., Heidt D.G., Dalerba P., Burant C.F., Zhang L., Adsay V., Wicha M.S., Clarke M.F., Simeone D.M. (2007). Identification of Pancreatic Cancer Stem Cells. Cancer Res..

[141] Momburg F., Roelse J., Howard J.C., Butcher G.W., Hämmerling G.J., Neefjes J.J. (1994). Selectivity of MHC-encoded peptide transporters from human, mouse and rat. Nat. Cell Biol..

[142] Zhang S., Balch C., Chan M.W., Lai H.-C., Matei D., Schilder J.M., Yan P.S., Huang T.H.-M., Nephew K.P. (2008). Identification and Characterization of Ovarian Cancer-Initiating Cells from Primary Human Tumors. Cancer Res..

[143] Buccarelli M., Marconi M., Pacioni S., De Pasqualis I., D’Alessandris Q.G., Martini M., Ascione B., Malorni W., LaRocca L.M., Pallini R. (2018). Inhibition of autophagy increases susceptibility of glioblastoma stem cells to temozolomide by igniting ferroptosis. Cell Death Dis..

[144] Kantara C., O’Connell M., Sarkar S., Moya S., Ullrich R., Singh P. (2014). Curcumin promotes autophagic survival of a subset of colon cancer stem cells, which are ablated by DCLK1-siRNA. Cancer Res..

[145] Han Y., Fan S., Qin T., Yang J., Sun Y., Lu Y., Mao J., Li L. (2018). Role of autophagy in breast cancer and breast cancer stem cells (Review). Int. J. Oncol..

[146] Gong C., Song E., Codogno P., Mehrpour M. (2012). The roles of BECN1 and autophagy in cancer are context dependent. Autophagy.

[147] Wolf J., Dewi D.L., Fredebohm J., Müller-Decker K., Flechtenmacher C., Hoheisel J., Boettcher M. (2013). A mammosphere formation RNAi screen reveals that ATG4A promotes a breast cancer stem-like phenotype. Breast Cancer Res..

[148] Sanchez C.G., Penfornis P., Oskowitz A.Z., Boonjindasup A.G., Cai D.Z., Dhule S.S., Rowan B.G., Kelekar A., Krause D.S., Pochampally R.R. (2011). Activation of autophagy in mesenchymal stem cells provides tumor stromal support. Carcinogenesis.

[149] Rahman A., Saha S.K., Rahman S., Uddin J., Uddin S., Pang M.-G., Rhim H., Cho S.-G. (2020). Molecular Insights Into Therapeutic Potential of Autophagy Modulation by Natural Products for Cancer Stem Cells. Front. Cell Dev. Biol..

[150] Altman B.J., Jacobs S.R., Mason E.F., Michalek R.D., MacIntyre A.N., Coloff J.L., Ilkayeva O., Jia W., He Y.-W., Rathmell J.C. (2010). Autophagy is essential to suppress cell stress and to allow BCR-Abl-mediated leukemogenesis. Oncogene.

[151] Karvela M., Baquero P., Kuntz E.M., Mukhopadhyay A., Mitchell R., Allan E.K., Chan E., Kranc K.R., Calabretta B., Salomoni P. (2016). ATG7 regulates energy metabolism, differentiation and survival of Philadelphia-chromosome-positive cells. Autophagy.

[152] Rothe K., Lin H., Lin K.B.L., Leung A., Wang H.M., Malekesmaeili M., Brinkman R., Forrest D.L., Gorski S.M., Jiang X. (2014). The core autophagy protein ATG4B is a potential biomarker and therapeutic target in CML stem/progenitor cells. Blood.

[153] Ojha R., Jha V., Singh S.K., Bhattacharyya S. (2014). Autophagy inhibition suppresses the tumorigenic potential of cancer stem cell enriched side population in bladder cancer. Biochim. Biophys. Acta (BBA) Mol. Basis Dis..

[154] Kenific C.M., Debnath J. (2014). Cellular and metabolic functions for autophagy in cancer cells. Trends Cell Biol..

[155] Chen J., Zhang L., Zhou H., Wang W., Luo Y., Yang H., Yi H. (2018). Inhibition of autophagy promotes cisplatin-induced apoptotic cell death through Atg5 and Beclin 1 in A549 human lung cancer cells. Mol. Med. Rep..

[156] Quan Y., Lei H., Wahafu W., Liu Y., Ping H., Zhang X. (2019). Inhibition of autophagy enhances the anticancer effect of enzalutamide on bladder cancer. Biomed. Pharmacother..

[157] Sheng B., Song Y., Zhang J., Li R., Wang Z., Zhu X. (2020). Atorvastatin suppresses the progression of cervical cancer via regulation of autophagy. Am J Transl Res.

[158] Shin D., Kim E.H., Lee J., Roh J.-L. (2017). RITA plus 3-MA overcomes chemoresistance of head and neck cancer cells via dual inhibition of autophagy and antioxidant systems. Redox Biol..

[159] Cheng X., Feng H., Wu H., Jin Z., Shen X., Kuang J., Huo Z., Chen X., Gao H., Ye F. (2018). Targeting autophagy enhances apatinib-induced apoptosis via endoplasmic reticulum stress for human colorectal cancer. Cancer Lett..

[160] Yuan N., Song L., Cai J., Wang J., Zhang Y., Mao X., Zhao W., Hu S., Chen S., Zhang S. (2015). Bafilomycin A1 targets both autophagy and apoptosis pathways in pediatric B-cell acute lymphoblastic leukemia. Haematologica.

[161] Lin J.-F., Lin Y.-C., Tsai T.-F., Chen H.-E., Chou K.-Y., Hwang T.I.-S. (2017). Cisplatin induces protective autophagy through activation of BECN1 in human bladder cancer cells. Drug Des. Dev. Ther..

[162] Li L.-Q., Xie W., Pan D., Chen H., Zhang L. (2016). Inhibition of autophagy by bafilomycin A1 promotes chemosensitivity of gastric cancer cells. Tumor Biol..

[163] Lin Y.-C., Lin J.-F., Wen S.-I., Yang S.-C., Tsai T.-F., Chen H.-E., Chou K.-Y., Hwang T.I.-S. (2017). Chloroquine and hydroxychloroquine inhibit bladder cancer cell growth by targeting basal autophagy and enhancing apoptosis. Kaohsiung J. Med Sci..

[164] Chung S.-F., Kim C.-F., Chow H.-Y., Chong H.-C., Tam S.-Y., Leung Y.-C., Lo W.-H. (2020). Recombinant *Bacillus caldovelox* Arginase Mutant (BCA-M) Induces Apoptosis, Autophagy, Cell Cycle Arrest and Growth Inhibition in Human Cervical Cancer Cells. Int. J. Mol. Sci..

[165] Scott E.C., Maziarz R.T., Cascio M.J., Podolak J., Gordon M., Botelho J., Stadtmauer E., Amaravadi R., Vogl D.T., Spurgeon S.E. (2017). Double autophagy stimulation using chemotherapy and mTOR inhibition combined with hydroxychloroquine for autophagy modulation in patients with relapsed or refractory multiple myeloma. Haematologica.

[166] Patel S., Hurez V., Nawrocki S.T., Goros M., Michalek J., Sarantopoulos J., Curiel T., Mahalingam D. (2016). Vorinostat and hydroxychloroquine improve immunity and inhibit autophagy in metastatic colorectal cancer. Oncotarget.

[167] Boone B.A., Bahary N., Espina V., Loughran P., Lotze M.T., Zeh H.J., Zureikat A.H., Moser A.J., Normolle D.P., Wu W.-C. (2015). Safety and Biologic Response of Pre-operative Autophagy Inhibition in Combination with Gemcitabine in Patients with Pancreatic Adenocarcinoma. Ann. Surg. Oncol..

[168] Egan D.F., Chun M.G., Vamos M., Zou H., Rong J., Miller C.J., Lou H.J., Raveendra-Panickar D., Yang C.-C., Sheffler D.J. (2015). Small Molecule Inhibition of the Autophagy Kinase ULK1 and Identification of ULK1 Substrates. Mol. Cell.

[169] Martin K.R., Celano S.L., Solitro A.R., Gunaydin H., Scott M., O’Hagan R.C., Shumway S.D., Fuller P., MacKeigan J.P. (2018). A Potent and Selective ULK1 Inhibitor Suppresses Autophagy and Sensitizes Cancer Cells to Nutrient Stress. iScience.

[170] Pasquier B. (2015). SAR405, a PIK3C3/Vps34 inhibitor that prevents autophagy and synergizes with MTOR inhibition in tumor cells. Autophagy.

[171] Dyczynski M., Yu Y., Otrocka M., Parpal S., Braga T., Henley A.B., Zazzi H., Lerner M., Wennerberg K., Viklund J. (2018). Targeting autophagy by small molecule inhibitors of vacuolar protein sorting 34 (Vps34) improves the sensitivity of breast cancer cells to Sunitinib. Cancer Lett..

[172] Luanpitpong S., Chanvorachote P., Nimmannit U., Leonard S.S., Stehlik C., Wang L., Rojanasakul Y. (2012). Mitochondrial superoxide mediates doxorubicin-induced keratinocyte apoptosis through oxidative modification of ERK and Bcl-2 ubiquitination. Biochem. Pharmacol..

[173] Yan C., Luo L., Guo C.-Y., Goto S., Urata Y., Shao J.-H., Li T.-S. (2017). Doxorubicin-induced mitophagy contributes to drug resistance in cancer stem cells from HCT8 human colorectal cancer cells. Cancer Lett..

[174] He L., Gu K. (2018). Tanshinone IIA regulates colorectal cancer apoptosis via attenuation of Parkin-mediated mitophagy by suppressing AMPK/Skp2 pathways. Mol. Med. Rep..

[175] Si L., Fu J., Liu W., Hayashi T., Mizuno K., Hattori S., Fujisaki H., Onodera S., Ikejima T. (2020). Silibinin-induced mitochondria fission leads to mitophagy, which attenuates silibinin-induced apoptosis in MCF-7 and MDA-MB-231 cells. Arch. Biochem. Biophys..

[176] Kocaturk N.M., Akkoc Y., Kig C., Bayraktar O., Gozuacik D., Kutlu O. (2019). Autophagy as a molecular target for cancer treatment. Eur. J. Pharm. Sci..

[177] Singh S.S., Vats S., Chia A.Y.-Q., Tan T.Z., Deng S., Ong M.S., Arfuso F., Yap C.T., Goh B.C., Sethi G. (2018). Dual role of autophagy in hallmarks of cancer. Oncogene.

[178] Russo M., Milito A., Spagnuolo C., Carbone V., Rosén A., Minasi P., Lauria F., Russo G.L. (2017). CK2 and PI3K are direct molecular targets of quercetin in chronic lymphocytic leukaemia. Oncotarget.

[179] Karpel-Massler G., Ishida C.T., Zhang Y., Halatsch M.-E., Westhoff M.-A., Siegelin M.D. (2017). Targeting intrinsic apoptosis and other forms of cell death by BH3-mimetics in glioblastoma. Expert Opin. Drug Discov..

[180] Takahashi A., Kimura F., Yamanaka A., Takebayashi A., Kita N., Takahashi K., Murakami T. (2014). Metformin impairs growth of endometrial cancer cells via cell cycle arrest and concomitant autophagy and apoptosis. Cancer Cell Int..

[181] Nazim U.M., Moon J.-H., Lee J.-H., Lee Y.-J., Seol J.-W., Eo S.-K., Lee J.-H., Park S.-Y. (2016). Activation of autophagy flux by metformin downregulates cellular FLICE-like inhibitory protein and enhances TRAIL- induced apoptosis. Oncotarget.

[182] Liu Y., Hao Y., Li Y., Zheng Y., Dai J., Zhong F., Wei W., Fang Z. (2020). Salinomycin induces autophagic cell death in salinomycin-sensitive melanoma cells through inhibition of autophagic flux. Sci. Rep..

[183] Bai Z., Ding N., Ge J., Wang Y., Wang L., Wu N., Wei Q., Xu S., Liu X., Zhou G. (2020). Esomeprazole overcomes paclitaxel-resistance and enhances anticancer effects of paclitaxel by inducing autophagy in A549/Taxol cells. Cell Biol. Int..

[184] Ozates N.P., Soğutlu F., Lerminoglu F., Demir B., Gunduz C., Shademan B., Avci C.B. (2020). Effects of rapamycin and AZD3463 combination on apoptosis, autophagy, and cell cycle for resistance control in breast cancer. Life Sci..

[185] Song L., Luo Y., Li S., Hong M., Wang Q., Chi X., Yang C. (2020). ISL Induces Apoptosis and Autophagy in Hepatocellular Carcinoma via Downregulation of PI3K/AKT/mTOR Pathway in vivo and in vitro. Drug Des. Dev. Ther..

[186] Boutouja F., Stiehm C.M., Platta H.W. (2019). mTOR: A Cellular Regulator Interface in Health and Disease. Cells.

[187] Blagosklonny M.V. (2012). Rapalogs in cancer prevention. Cancer Biol. Ther..

[188] Wang H., Li D., Li X., Ou X., Liu S., Zhang Y., Ding J., Xie B. (2016). Mammalian target of rapamycin inhibitor RAD001 sensitizes endometrial cancer cells to paclitaxel-induced apoptosis via the induction of autophagy. Oncol. Lett..

[189] Dai Z., Gao J., Ma X., Kang H.-F., Wang B., Lu W.-F., Lin S., Wang X.-J., Wu W.-Y. (2012). Antitumor Effects of Rapamycin in Pancreatic Cancer Cells by Inducing Apoptosis and Autophagy. Int. J. Mol. Sci..

